# A novel method in the removal of impacted mandibular third molar: buccal drainage

**DOI:** 10.1038/s41598-017-12722-8

**Published:** 2017-10-03

**Authors:** Ting Hu, Ji Zhang, Jing zhi Ma, Le nan Shao, Yi fei Gu, Dian qi Li, Liang Jiang, Yun qiang Yang

**Affiliations:** 1 Department of Stomatology, Tongji Hospital, Tongji Medical College, Huazhong University of Science and Technology, Wu han, 430030 China; 2 Department of Obstetrics and Gynecology, Tongji Hospital, Tongji Medical College, Huazhong University of Science and Technology, Wu han, 430030 China; 3 Department of Neurosurgery, State Key Laboratory of Oncology in South China, Sun Yat-sen University Cancer Center, Collaborative Innovation Center for Cancer Medicine, Guang zhou, 510060 China; 4 Dental Implant Center, State Key Laboratory of Oral Diseases, West China College of Stomatology, Sichuan University, Chengdu, 610041 China

## Abstract

Food impaction after impacted mandibular third molar extraction is a serious problem that should not be ignored. Incomplete suturing of the distal incision in the conventional method is the main cause of food impaction and delayed wound healing. The present study introduces a novel suture and drainage technology that requires hermetic suturing of the distal incision and rubber drainage for buccal drainage. 76 patients with horizontally/mesially impacted third molars (bilateral) were enrolled in this prospective study. An impacted tooth on one side of each patient was extracted by occlusal drainage using the conventional method, whereas the other side tooth was extracted by buccal drainage using the novel method. The differences in wound healing, facial swelling, bleeding and dry socket between the two sides of each patient were compared postoperatively, and the trends for patient selection of the surgical method were also compared. The results indicated that buccal drainage had obvious advantages in wound healing and reduced the risk of postoperative bleeding, and most patients preferred this technique; there were no significant differences in postoperative facial swelling or pain. Thus, buccal drainage can solve the problem of long-term food impaction induced by traditional incision postoperatively and is worthy of clinical promotion.

## Introduction

Impacted teeth are most commonly found among mandibular third molars and lead to clinical diseases including pericoronitis, damage to adjacent teeth and temporomandibular disorders^1^. Impaction is also a potential cause of odontogenic cysts and tumours. Therefore, mandibular third molars are often extracted. Among the many types of mandibular third molar impaction, horizontally and mesioangularly impacted teeth are the most difficult to extract, regardless of the exceptional cases^2,3^. Complications may occur after extraction. Permanent nerve damage and serious infections are the most severe complications, but the rate of such complications is low^4,5^. Pain, trismus, dry socket and swelling are the most common postoperative complaints and influence the patients’ quality of life in the days^1,6^.

Suturing is necessary during the extraction of impacted mandibular third molars and is aimed at recovering the soft tissues for better healing. Furthermore, suturing reduces postoperative bleeding and narrows the extraction socket, which can prevent food from entering into it and protect the blood clots^7^. The wound should not be sutured too firmly, and the turning point of the incision, which is adjacent to the distal side of the second molar, is often left without stitching, setting up a drainage system through the occlusal surface between the distal side of the second molar and the mesial side of the primary third molar^8^. Many experts believe that the creation of such a drainage pathway for inflammatory exudates helps to reduce facial swelling, pain and other postoperative complications^8–10^. The main drawback of sutureless techniques is that wound healing may be delayed. In addition, there may be a high potential for the formation of a wedge gap in relation to the adjacent second molar, which may cause food impaction^7,8^. We also found that patients treated with such a drain system often complain that they are unwilling to chew with the affected side because of concerns that food will enter the extraction socket. The disto-occlusal region with this type of drainage system takes much longer to heal than does the maxilla, and patients are extremely uncomfortable^8,11^. Actually, food more easily gets into the mandibular extraction wound under gravity, compared with the maxillary wounds. Thus, food impaction is a serious clinical problem after the mandibular impacted third molar extraction, except for pain, trismus, dry socket and other common postoperative complaints, and is worthy of attention. However, few publications refer to this situation, and doctors seldom discuss how to solve this problem.

Consequently, we propose a new set of suture and drainage methods, named buccal drainage. The aim of the study is that such a method can solve the long standing problem of food impaction and delayed wound healing and does not increase the risk of relevant postoperative complications.

## Materials and Methods

### Study Samples

To address the research purpose, a prospective study was conducted. Patients with horizontal/mesioangular impacted mandibular third molars who visited the Department of Stomatology of Tongji Hospital from 2014.08 to 2016.08 were included in this study.

Inclusion criteria: ① The molars were horizontally/mesioangularly impacted on both sides, and all teeth were partially or completely covered by mucosa; ② The region, size and resistance distribution of the bilaterally impacted molars were similar; ③ The patients were in good medical condition, without local inflammation; ④ All patients were treated with the traditional method on one side and the new technique on the other side; ⑤ All patients had complete medical records and follow-up records, and they could clearly remember the relevant complications after the tooth extraction. ⑥ This is a double blind study with the patients have no knowledge at which site is which.

Our study was approved by the Ethics Committee of Huazhong University of Science and Technology, and all of the patients signed informed consent forms. All methods were performed in accordance with the relevant guidelines and regulations.

### Anaesthesia Method

All patients received one-time block anaesthesia in the inferior alveolar nerve, lingual nerve and buccal nerve using a 2% lidocaine hydrochloride (1:200,000 epinephrine) anaesthetic solution. Terminal infiltration of the buccal fold and distal incision region was also performed using primacaine with adrenaline.

### Surgical method

One surgeon operated on all cases. All patients had their impacted molars on one side extracted with the traditional method, and after 1~4 weeks, they had their impacted molars on the other side extracted with the new method. All of the incisions were designed as a triangular flap.

#### Traditional triangular incision (controlled group)

**Figure 1:**
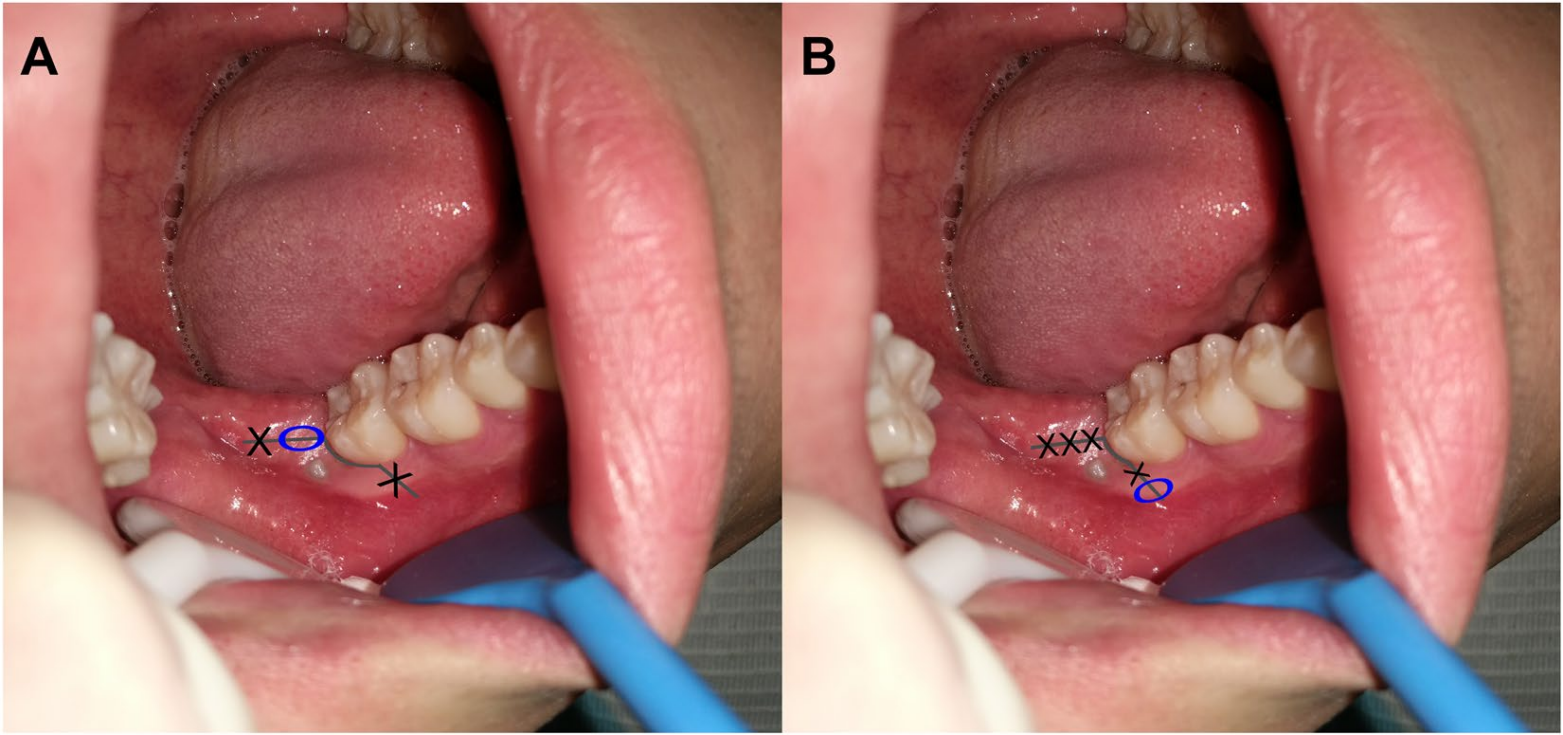
The incision and suture schematic diagram used in impacted mandibular third molar. figure A: conventional method, partially suture the distal incision and the oval area indicate the occlusal drain pathway; figure B:new method, firmly suture the distal incision and the oval area indicate the buccal drain pathway which is also the place for drainage strip throughout, and the mesio-incision derived from the distal of the second molar.

The mesio-buccal incision started from the mesio-buccal or disto-buccal axial angles of the adjacent tooth and was at a 45° angle with the gingival margin. The incision moved downward and forward but did not surpass the bottom of the transitional channel. The distal incision started from the middle point of the second molar’s distal gingival margin and extended postero-laterally. The incision did not deviate to the lingual side and was long enough to expose the buccal and distal bone surfaces after flap surgery. Then, the molar mesioangular dental crowns were split with dental high-speed turbine handpieces. In suturing, we ordinarily placed one stitch in the mesio-buccal incision and another stitch in the distal incision, and we added one more stitch in both regions if necessary. The mesio-occlusal region of the third molar was left unstitched for drainage (Fig. 1).

#### New incision for buccal drainage (experimental group)

**Figure 2:**
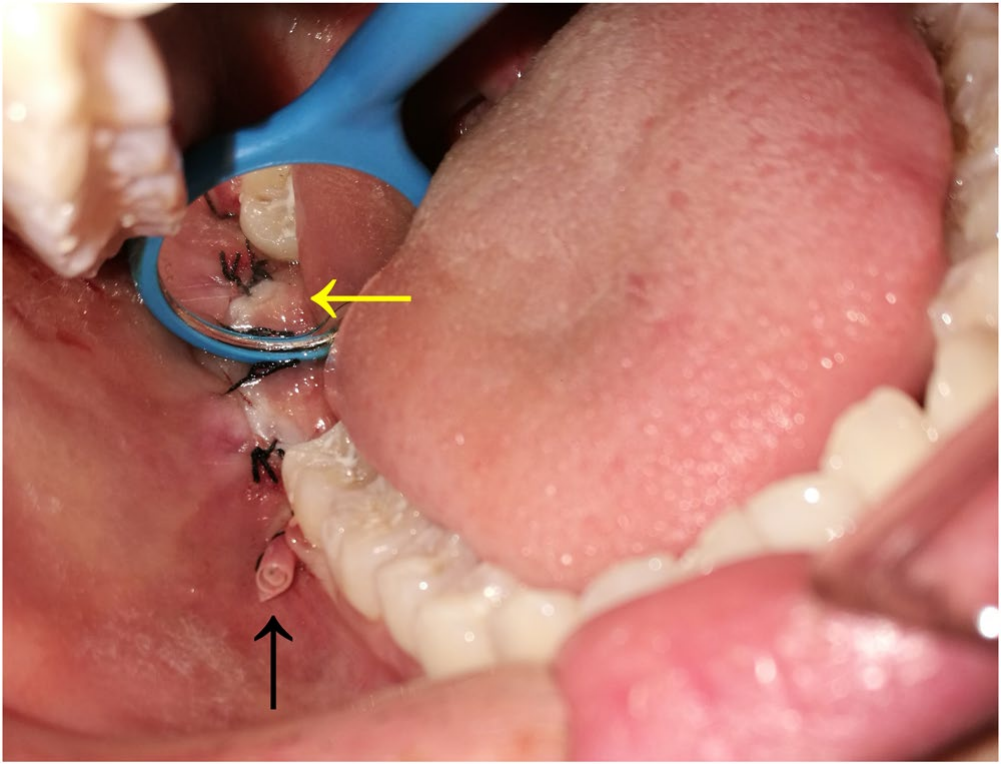
The diagram of the new technique for suture and drainage in the removal of impacted mandibular third molar: the yellow arrow indicates firmly suturing the distal incision from odontoscope; the black arrow indicates the buccal drain strip.

The mesio-buccal incision started from the disto-buccal aspect of the second molar region, and not the mesio-buccal region of the adjacent tooth, for successful drainage. Its distal incision was equal to that in the traditional method. In suturing, we first firmly stitched the distal incision with at least two stitches. The strip emerged from the buccal incision, which was only sewed for 1~2 stitches on two sides to fix the strip (Figs 1 and 2).

### Postoperative follow-up

All patients iced their faces and cheeks discontinuously during the 24 hours after the operation and took cephalosporin and metronidazole for 3~5 days after the operation; most of the patients also took NSAIDs after the operation. All patients’ drainage strips were removed 72 hours after surgery, and their stitches were removed 7 days after the operation. We paid follow-up visits to all patients on the 1st day, 3rd day, and 7th day as well as 1 month and 3 month after the operation.

### Observational index

We made horizontal comparisons for each patient at the same timepoints, to observe the differences in facial swelling, bleeding, and dry socket between the two sides of the same patient. We recorded their wound healing and food impaction statements at 1~3 months after the operations, as well as their preferences regarding the surgical methods. All observational indexes were determined on the basis of the combination of clinical observations and patients’ subjective assessments.

The comparison of bleeding was accomplished within 7 days, and the main comparative contents were as follows: ① Whether there was active bleeding during the first day after surgery, especially the several hours after the cotton balls were spat out. ② Whether there was active bleeding during the second and third day after surgery. ③ The comparison of ecchymosis on the cheek and submandibular region. Patients were usually sensitive to postoperative bleeding, and through the three methods, we could determine the differences between the two sides.

The facial swelling assessments were also accomplished within 7 days. The patients took photos of their face on the first, third and seventh day during the follow-up visits. The differences in facial swelling bilaterally were evaluated mainly through subjective assessments by the patients and through photo comparisons.

The comparison of food impaction and wound healing was accomplished in 3 months. In the clinical study, the patients reported food impaction based on subjective feelings, which could not be quantified, whereas the doctor evaluated this complication through wound healing. The patients ceased to feel food impaction only when the disto-occlusal incisions were completely healed. These observation indexes were rated and compared at 7 days, 1 month and 3 months, respectively. Food impaction is the clinical manifestation of incomplete wound healing; thus, we used wound healing to replace food impaction in the following research.

### Statistical analysis

We used SPSS 13.0 software (IBM, Armonk, NY) to conduct a chi-square test. The data were statistically significant when P < 0.05. Because of the special design in this research, we compared the differences in the ratios of the patients’ preferences between the new method and the conventional method among different postoperative complications and surgical types.

## Results

In total, 76 individuals were included in this study. Their age varied between 19 and 35 years, with an average of 25.6 years. The number of impacted mandibular third molars in these patients was 152.

The new method had obvious advantages in wound healing. Seventy-two teeth were firmly stitched in the occlusal surface 1 week after the operation, and 4 teeth were not quite firmly stitched because of certain reasons, including suturing problems. All wounds treated with the new method healed 1 month after the operation. The distal parts of the second molar on the control sides showed wedged gaps of different sizes after 1 month postoperatively; only a few of them healed completely, and some of them still had not healed even half a year after the operation.

Regarding postoperative facial swelling, 57 of the patients showed no differences between the two sides; the ratio was 57/76. Eleven of them believed the traditional incisions were better, with less facial swelling, whereas 8 patients preferred the new method. The differences in the ratio of facial swelling between the new method and the conventional method were not significant (P > 0.05).

Regarding postoperative bleeding, no patients exhibited massive haemorrhage after the operation. Twenty patients said they bled less with the new method, and 4 patients supported the traditional method. Fifty-two patients found no differences between the two methods. The differences in the ratio of bleeding between the new method and the conventional method showed obvious statistical significance (P < 0.001).

Only 2 patients exhibited suspected dry socket on the control side after the operation and recovered quickly after appropriate treatment. As to trismus, all of the patients had nearly normal opening mouth after 7 days. None of the patients suffered nerve injury and evident pain in this study.

**Figure 3:**
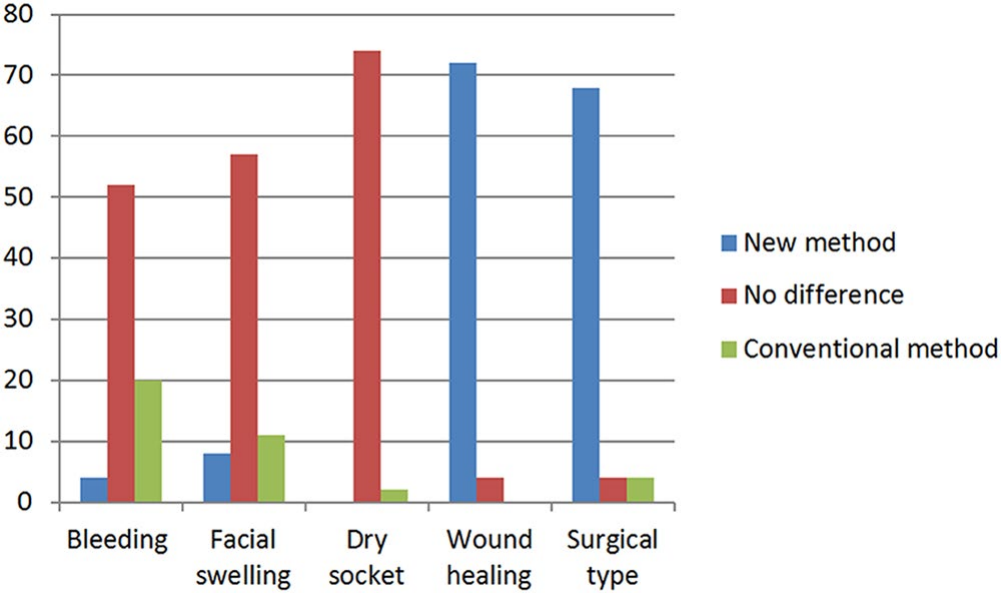
The statistical chart of patients’ viewpoint and choice in postoperative complication and surgical type. each patient has three choice (new method, no difference and conventional method).

Finally, 68 patients supported the new method and were concerned about food residues in the distal part of the traditional incisions and feelings of discomfort. Four patients found no differences between the two methods, and only 4 patients preferred the traditional method. The results are shown in Fig. 3 and Table 1.

**Table 1:** Patients’ choices regarding postoperative complications and surgical type.

	Bleeding	Dry socket	Facial swelling	Wound healing	Surgical type
New method(ratio)	24/76	00	88/76	372/76	368/76
Conventional method(ratio)	20/76	2/76	11/76	0	4/76
*P* value	<0.001	0.50	0.62	<0.001	<0.001

## Discussion

There are many types of complications after impacted tooth extraction, including the commonly known bleeding, infection, nerve injury and dry socket. Furthermore, patients can feel quite uncomfortable postoperatively, and they dare not use the affected side to chew, due to concerns regarding food entering the extraction wound^8^. This situation occurs because the traditional drainage system is placed in the disto-occlusal region of the second molar, which causes an incompletely healed wedged gap for a long period. Food impaction disappears only when the wound is completely healed. Food impaction is simply a clinical manifestation of incomplete wound healing, especially with respect to the disto-occlusal incision. In our study, we transferred the drainage channel from the occlusal surface to the buccal surface, which improved the healing effect of the disto-occlusal incision and prevented food from entering. In this way the chewing function on the affected side could recover as soon as possible, and the patients felt comfortable after the operations, without experiencing food impaction.

There are many styles of flap design in the removal of impacted mandibular third molars; however, there are very few specific data available from the literature regarding postoperative flap-related discomfort. Grossi *et al*. confirmed that the triangular flap design is associated with patients consuming the least painkillers^2^. Thus, we adopted the triangular flap for each impacted molar extraction, which was also widely used clinically. The traditional method drains from the gap on the distal region of the second molar, and the draining effect is good because the gap is usually wide. The most concerning problem of the new method is postoperative swelling, and we found no difference during clinical observations. We believe that is because of the advantage of the drainage posture. The drainage strip is placed under the dental crown, and such a posture is good for effective drainage under gravity and make ups the shortage of the drainage gap compared with the traditional method. Specially, the buccal incision of the new method must be performed in the distal region of the second molar, preferably clinging to the extraction wound, which can maximum the drainage effect. If the buccal incision is not placed in the distal region of the adjacent molar, then a portion of the drainage strip will cling to the buccal bone surface of the mandible, which will lengthen the drainage distance, narrow the drainage space, and reduce the drainage effect.

Relevant clinical research already exists. Bello *et al*. reported lower swelling in the group with a partial closure of the wound compared with complete closure of the wound, and they did not find differences regarding trismus or pain^8^. Maria *et al*. found a lower level of postoperative variables in the group with a secondary closure, as well as a greater level of oedema and the presence of haematomas in the group with a complete closure^12^. Such research confirms that a drain is imported in impacted tooth extraction and that completely suturing the incision without a suitable drainage pathway should not be adopted clinically, which was the same as our clinical experience. However, some researchers have evaluated the role of the suture technique in relation to postoperative complications and concluded that there are no significant differences between complete closure and a one-knot in the corner of the buccal incision of the triangular flap, which uses the buccal relief incision for drainage without a drain strip^7,13^. Many experts have confirmed that the use of a drain strip can reduce postoperative facial swelling and has no effect on pain or trismus^14–16^. Because the gap between the buccal flap and bone surface is extremely narrow even though the buccal incision was partially sutured when there was no use of a drain strip, we believe the partial suturing of the buccal-relieving incision had no evident effect on swelling compared with completely suturing, which can also explain the results we mentioned above. The core of our research was to change the drainage pathway from the occlusal surface to the buccal surface using a drain strip, which can create an adequate gap between the buccal flap and the bone surface for drainage.

The new method showed better haemostasis than did the traditional method because of the firm occlusal suture. Four patients preferred the traditional method, and we believe that was because their incisions extended to the bottom of the vestibular groove and the extraction difficulty of the control side was obviously greater, as impacted molars on two different sides do not always present in a similar fashion. The buccal incisions were not firmly sutured and therefore bled much, which reminds us that we should not make too-long buccal incisions, which are not allowed to surpass the bottom of the vestibular groove.

Regarding dry socket, we did not find a significant difference between the two methods because of the limited number of cases. At present, we must reduce the surgery trauma, protect the blood clot, maintain oral hygiene and provide full rest postoperatively, to prevent dry socket^6^. By using the new method, we can maximally protect the blood clot and prevent external irritants with a firmly occlusal suture. We believe that the new method can reduce the incidence of dry socket compared with the traditional method in large samples.

According to our study, most patients were willing to use the new method. Only 4 patients held an adverse opinion. The inexperience of the doctor in using the new method was the main reason for patients who opposed the new method, and these patients also believed that the new method took too much longer to place the drainage strip compared with the traditional method, and the traditional incisions also healed well for a period of time. In the future, we need to find the most appropriate drainage device to reduce the operating time and improve the clinical result.

Above all, the new method can solve the problem of a long-standing wedged gap in the disto-occlusal region of the second molar, make patients feel better, and reduce the incidence of postoperative bleeding and dry socket. Therefore, this technique is worthy of clinical promotion.
